# Electrocardiography versus photoplethysmography in assessment of maternal heart rate variability during labor

**DOI:** 10.1186/s40064-016-2787-z

**Published:** 2016-07-15

**Authors:** Hernâni Gonçalves, Paula Pinto, Manuela Silva, Diogo Ayres-de-Campos, João Bernardes

**Affiliations:** Center for Health Technology and Services Research (CINTESIS), Faculty of Medicine, University of Porto, Rua Dr Plácido da Costa, s/n, 4200-450 Porto, Portugal; Department of Obstetrics and Gynecology, Medical School, University of Porto, Porto, Portugal; Hospital Dr Nélio Mendonça, EPE, Funchal, Portugal; Department of Obstetrics and Gynecology, São João Hospital, Porto, Portugal; INEB - Institute of Biomedical Engineering, Porto; I3S - Institute for Research and Innovation in Health, University of Porto, Porto, Portugal; Hospital Pedro Hispano, Unidade Local de Saúde de Matosinhos, Senhora da Hora, Portugal

**Keywords:** Maternal heart rate, Fetal monitoring, Cardiotocography, Signal processing, Electrocardiography, Pulse oximetry

## Abstract

**Purpose:**

Evaluation of maternal heart rate (MHR) variability provides useful information on the maternal-fetal clinical state. Electrocardiography (ECG) is the most accurate method to monitor MHR but it may not always be available, and pulse oximetry using photoplethysmography (PPG) can be an alternative. In this study we compared ECG and PPG signals, obtained with conventional fetal monitors, to evaluate signal loss, MHR variability indices, and the ability of the latter to predict fetal acidemia and operative delivery.

**Methods:**

Both signals were simultaneously acquired in 51 term pregnancies during the last 2 h of labor (H_1_ and H_2_). Linear time- and frequency-domain, and nonlinear MHR variability indices were estimated, and the dataset was divided into normal and acidemic cases, as well as into normal and operative deliveries. Differences between ECG and PPG signals were assessed using non-parametric confidence intervals, hypothesis testing, correlation coefficient and a measure of disagreement. Prediction of fetal acidemia and operative delivery was assessed using areas under the receiver operating characteristic curve (auROC).

**Results:**

Signal loss was higher with ECG during the first segments of H_1_, and higher with PPG in the last segment of H_2_, and it increased in both signals with labour progression. MHR variability indices were significantly different when acquired with ECG and PPG signals, with low correlation coefficients and high disagreement for entropy and fast oscillation-based indices, and low disagreement for the mean MHR and slow oscillation-based indices. However, both acquisition modes evidenced significant differences between H_1_ and H_2_ and comparable auROC values were obtained in the detection of fetal acidemia and operative vaginal delivery.

**Conclusion:**

Although PPG captures the faster oscillations of the MHR signal less well than ECG and is prone to have higher signal loss in the last 10-min preceding delivery, it can be considered an alternative for MHR monitoring during labor, with adaptation of cut-off values for MHR variability indices.

## Background

Computerized analysis of maternal heart rate (MHR) recordings obtained by electrocardiography (ECG) may help in the assessment of different clinical maternal-fetal conditions, during the antepartum period (DiPietro et al. 2003; Lao et al. 2009; Pinto et al. 2014; Söhnchen et al. 2011; Tejera et al. 2011; Van Leeuwen et al. 2009; Weissman et al. 2009) and in the detection of MHR-fetal heart rate (FHR) ambiguities during labor (Bernardes and Ayres-de-Campos 2012; Hanson 2010; Murray 2004; Sherman et al. 2002).

In clinical practice, however, it is not always possible to obtain MHR recordings with ECG during labour, because many fetal monitors do not incorporate this technology, and some healthcare professionals and laboring women think it is unnecessary and interferes with the physiological experience of childbirth. Alternatively, MHR can be obtained with a pulse oximetry sensor and photoplethysmography (PPG), using monitors that have been integrated or are coupled to fetal monitors. Continuous monitoring of maternal oxygen saturation is required in some clinical situations during labor, and some women find it more comfortable than chest electrodes. Moreover, some fetal monitors have recently incorporated pulse oximetry into the tocodynomometer sensor, so MHR can be obtained with no extra equipment. However, there is no data on whether MHR analysis is equivalent when obtained with ECG or PPG.

MHR acquired either by ECG or PPG is transmitted to fetal monitors, which store FHR and MHR simultaneously, typically at a same regular time basis, by means of signal interpolation (STAN Service Manual 2005). However, the heart rate (HR) obtained with PPG is less accurate than that acquired with ECG (Lu and Yang 2009), particularly during exercise (Iyriboz et al. 1991). Similarly, this may also happen in other situations of increased physical effort, namely during labor.

The accuracy of PPG appears to be suitable for the analysis of the HR of newborn infants in the delivery room (Kamlin et al. 2008), in the neonatal intensive care unit (Singh et al. 2008), and in the study of obstructive sleep apnea syndrome, using spectral analysis (Zamarrón et al. 2001) or entropy methods (Hornero et al. 2007), or in the detection of FHR decelerations, in the intrapartum period (Puertas et al. 2005). In addition, artifacts that contaminate PPG waveforms can be automatically rejected using methods based on waveform morphology analysis (Sukor et al. 2011).

To date, no studies have evaluated whether PPG is as accurate as ECG for MHR acquisition during labour. In addition, no studies have compared signal loss when using the two methods.

The objective of this study was to compare simultaneously-acquired ECG and PPG signals for the detection of MHR rate during labour. The following parameters were evaluated in both signals: signal loss, variability indices as evaluators of signal characteristics, and the ability of the latter to predict fetal acidemia and operative delivery. The rationale for MHR to be able to predict fetal acidemia and operative delivery, comes from the knowledge that many situations of fetal hypoxia/acidosis and abnormal labor progression (with its surrogate indicator of operative delivery) are associated with an anomalous pattern of uterine contractions (Ayres-de-Campos et al. 2015), and the latter is related with maternal sympatho-vagal activity (Kovács et al. 2015; Nagel et al. 2014). MHR variability could thus provide an early sign of fetal academia and/or labour dystocia, by means of the autonomic changes associated with altered uterine contraction dynamics.

## Methods

### Data acquisition

A total of 51 MHR recordings, pertaining to 51 different laboring women, were obtained simultaneously with ECG and PPG during the last 2 h before delivery (H_1_ and H_2_). All signals were acquired in uneventful singleton term pregnancies, and all women were under epidural analgesia. The study was approved by the hospital’s Ethics Committee (“Parecer no. 19/08, Comissão de Ética para a Saúde do Serviço Regional de Saúde, E.P.E.”) and all mothers gave informed consent to participate.

In order to assess the capacity of ECG and PPG signals in the classification of fetal acidemia and operative vaginal delivery, the dataset was divided into normal and acidemic groups—considering an umbilical artery blood (UAB) pH threshold of 7.15—and into normal and operative vaginal deliveries. The main maternal and perinatal characteristics of the study groups are presented in Table 1.

**Table 1:** Main maternal and perinatal characteristics of the study population, with the subdivisions into normal versus acidemic groups, and normal versus operative vaginal deliveries

	Fetuses born with UAB pH ≥7.15 (n = 45)	Fetuses born with UAB pH <7.15 (n = 6)	p-value	Normal deliveries (n = 28)	Vaginal operative deliveries (n = 19)	p-value
Maternal data, median (IQR)						
Age (years)	28 (3.5)	27 (10)	0.597	28 (8.0)	28 (9.0)	0.550
Parity	0 (1)	0 (1)	0.853	0 (1)	0 (0)	*0.001*
Gestational age (weeks)	39.9 (1.3)	39.8 (2.5)	0.578	39.7 (1.1)	39.9 (1.1)	0.415
Delivery, n (%)			0.693			–
Vaginal, normal	25 (56 %)	3 (50 %)		–	–	
Vaginal, operative	17 (37 %)	2 (33 %)		–	–	
Cesarean section	3 (7 %)	1 (17 %)		–	–	
Epidural analgesia, n (%)	45 (100 %)	6 (100 %)	–	28 (100 %)	19 (100 %)	–
Newborn data, median (IQR)						
Birthweight (g)	3190 (393)	3078 (381)	0.255	3160 (350)	3200 (405)	0.871
1 min Apgar score	9 (1)	9 (0)	0.679	9 (1)	9 (1)	0.957
5 min Apgar score	10 (0)	10 (0)	0.679	10 (0)	10 (0)	0.687
UAB pH	7.27 (0.09)	7.10 (0.06)	*0.000*	7.27 (0.10)	7.22 (0.09)	0.109
Gender, n (%)			0.191			0.246
Males	24 (53 %)	1 (17 %)		11 (39 %)	11 (58 %)	
Females	21 (47 %)	5 (83 %)		17 (61 %)	8 (42 %)	

Values within brackets in the header correspond to the number of cases

p-value <0.05 is in italic

MHR obtained with ECG was acquired with two unipolar chest electrodes placed on three classical locations (2nd right and left intercostal spaces and 5th left intercostal space in the medioclavicular line) linked to a conventional STAN^®^31 fetal monitor (Neoventa, Gothemburg, Sweden). The monitor amplifies the signals, digitalises them at a sample rate of 1600 Hz with a 12-bit precision, and then applies a filter. A heart period is calculated from the RR interval and converted to the nearest integer, in beats per minute (STAN Service Manual 2005).

MHR obtained by PPG was acquired using a conventional pulse oximetry infrared finger probe, linked to the VitalCare 506DN3 Vital Signs Monitor (Criticare Systems, Inc., Wisconsin, USA), which was connected to the STAN^®^ 31 fetal monitor. The HR period is derived from analysis of the dual wavelength LED waves, representing arterial blood volume changes, and is transmitted to the fetal monitor at a sampling rate of 1 Hz with an accuracy and resolution of ±1 bpm (Criticare Systems, Inc. 2009).

**Fig. 1:**
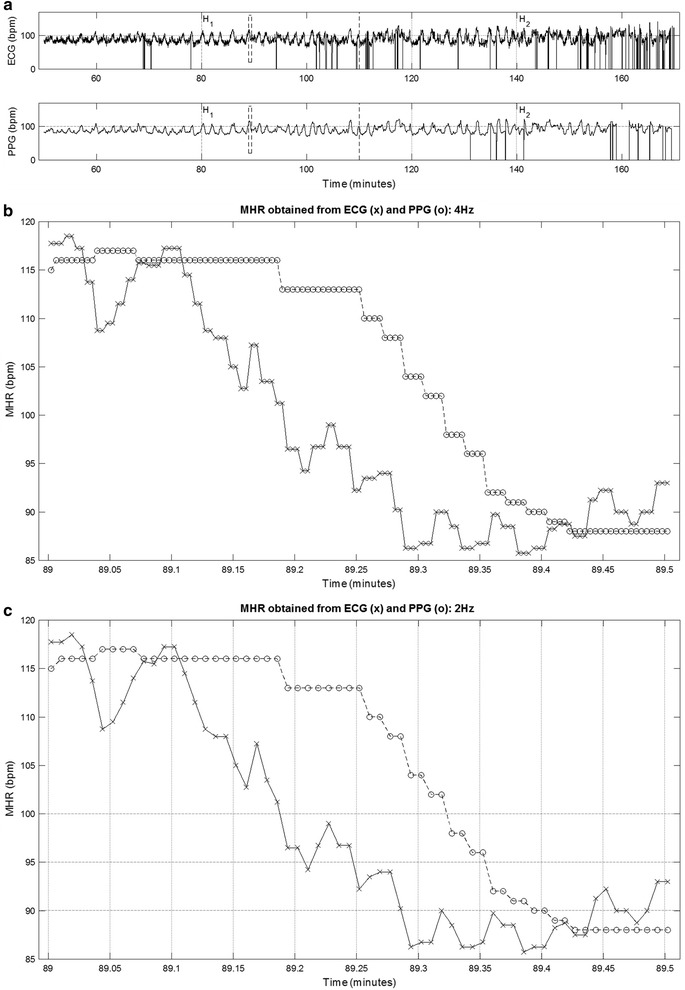
An example of a MHR tracing obtained with ECG (*upper plot*) and PPG (*lower plot*) for H_1_ and H_2_ (**a**). The* rectangle* between* vertical dashed lines* in **a** represents the 30-s segment shown in **b** and **c**, obtained respectively at 4 and 2 Hz sampling rates, with ECG (*marked with a cross*) or PPG (*marked with a circle*) signals

Both ECG and PPG signals were exported from the STAN monitor at a sampling rate of 4 Hz, via its RS232 port, into the Omniview-SisPorto^®^ 3.5 program (Speculum, Lisbon, Portugal) for storage and subsequent offline analysis. After the application of a pre-processing algorithm described by Gonçalves et al. (2006a), with an adaptation to scale, the signals were resampled at a frequency of 2 Hz considering only the odd samples, which mitigates the repetition of MHR values, keeping them below the Nyquist frequency (the spectrum of interest is ≤0.4 Hz). Figure 1 displays an example of MHR signals simultaneously acquired by ECG and PPG, where it is patent that the PPG signal smoothens the abrupt oscillations occurring in very short time periods, and thus delays baseline shifts, although this is compensated afterwards.

### MHR analysis

MHR recordings were evaluated using linear (time- and frequency-domain) and nonlinear methods, applied to 10-min segments. The segment-based analysis mitigates the possible effect of non-stationarity. Segments with more than 15 % signal loss (the percentage of MHR values equal to 0) before the pre-processing operation, in one or both of the acquisition modes (ECG or PPG), were excluded from MHR variability analysis.

For time domain linear analysis, the following indices were calculated: mean MHR (mHR); standard deviation of MHR (sdHR); long-term irregularity index, assessed by the inter-quartile range of the squared root of the sum of consecutive pairs of squared samples (LTI); Delta MHR representing the average amplitude within a minute (Δ); short-term variation (STV); and interval index (II). All but II reflect gross changes in MHR average and variability, whereas II assesses short-term MHR variability taking into account long-term variability (Gonçalves et al. 2006a). For frequency domain analysis, the following frequency bands were considered: very low frequency (VLF) at 0–0.04 Hz, low frequency (LF) at 0.04–0.15 Hz and high frequency (HF) at 0.15–0.40 Hz (Task force of the European Society of Cardiology, the North American Society of Pacing and Electrophysiology 1996). Nonparametric spectrum estimation was performed according to a procedure previously described in detail (Gonçalves et al. 2006a). The whole spectrum area corresponds to the total power (TP). The VLF band appears to reflect thermoregulatory and slow regulating systems of peripheral vessels (Task force of the European Society of Cardiology, the North American Society of Pacing and Electrophysiology 1996) and occurs prominently when autonomic activity is strongly suppressed. LF is associated with the activity of both the sympathetic and parasympathetic branches of the autonomic nervous system, whereas HF is mainly associated with the parasympathetic branch. LF_norm_ and HF_norm_ were also computed by normalizing each absolute value by TP-VLF. The LF/HF index, which reflects the balance between the autonomic nervous system branches, was also considered.

For non-linear analysis, approximate entropy (ApEn) (Pincus 1991), and sample entropy (SampEn) (Richman and Moorman 2000) were calculated, considering values 0.1 SD, 0.15 SD and 0.2 SD for r and value 2 for m (Pincus and Viscarello 1992), while N was 1200 points (corresponding to 10-min MHR segments). The criterion for selection of the threshold parameter r proposed by Lu et al. (2008) was also considered. Entropy indices have been associated with complex cortical nervous system activity (Pincus and Viscarello 1992). The Poincaré plot is one of the most popular techniques for analysis of heart rate variability (HRV) (Acharya et al. 2006). It is formed from the representation of each RR interval against its previous RR interval. The most commonly used HR variability indices based on the Poincaré plot are SD1, SD2, and the ratio SD1/SD2, where SD1 and SD2 are related to fast beat-to-beat and longer-term variability, respectively (Acharya et al. 2006). These three nonlinear measures were also considered, and the Poincaré plot was constructed.

### Statistical analysis

The ECG and PPG signals were compared by evaluating the percentage of signal loss, the percentage of MHR values obtained from ECG and PPG differing less than or equal to 5 bpm (HR_m_) (Behar et al. 2014; Association for the Advancement of Medical Instrumentation 2002) and the previously described MHR variability indices in the six 10-min segments of H_1_ and H_2_. Statistical inference was based on 95 % bootstrap (B = 1000) percentile confidence intervals for the median, Spearman correlation of coefficient and nonparametric Mann–Whitney statistical test with significance level set at p < 0.05 (Dudewicz and Mishra 1998; Martinez and Martinez 2002). Disagreement was assessed through the information-based approach dAB (Costa-Santos et al. 2010), ranging between 0 and 1 (from lowest to highest disagreement). The ability of each MHR variability index to predict newborn acidemia and operative vaginal delivery was determined using areas under receiver operating curve (auROC).

## Results

**Fig. 2:**
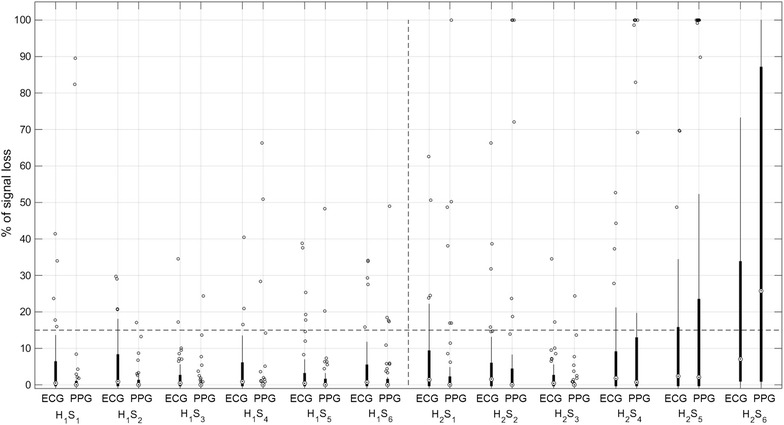
Percentage of signal loss with ECG and PPG, across all the 10-min segments S_i_ (i = 1,…,6) of H_1_ and H_2_, represented as *boxplots*. The *horizontal dashed line* corresponds to the 15 % signal loss threshold used to exclude segments from analysis of MHR variability indices

The percentage of MHR signal loss for the ECG and PPG signals increased as labor progressed, from H_1_ to H_2_, as displayed in Fig. 2. Median signal loss with ECG was significantly higher than with PPG in the first four segments of H_1_ (1 vs 0 %), exhibiting a pronounced positively skewed distribution (Fig. 2). Median signal loss with PPG was significantly higher than with ECG in the last segment of H_2_ (10 vs 20 %). A total of 38 out of 306 segments in H_1_ (12.4 %) and 113 out of 306 segments in H_2_ (36.9 %) were excluded hereafter from analysis due to signal loss higher than 15 % in ECG and/or PPG signals (Fig. 2). MHR values differing less than or equal to 5 bpm between ECG and PPG signals (HR_m_), had a median of 76.4 % (inter-quartile range of 17.5 %) in H_1_, and 71.5 % (inter-quartile range of 22.6 %) in H_2_. An episode with differences higher than 5 bpm can be observed in Fig. 1.

MHR variability indices obtained from PPG signals were significantly lower than those obtained from ECG signals, with the exception of LF_norm_ and LF/HF (Table 2). The lower values of short-term variability and entropy indices are likely to be due to the inability of PPG to capture faster signal oscillations, as showed in Fig. 1. Most linear indices and SD_2_ obtained with the two methods were highly correlated, with values ranging between 0.60 and 1.00, whereas LF_norm_, HF_norm_, LF/HF and the remaining non-linear indices had lower correlations, ranging between 0.07 and 0.59 (Table 2).

**Table 2:** MHR variability indices, obtained with ECG and PPG in H_1_ and H_2_, presented as 95 % confidence intervals (95 % CI), with the corresponding p-values and correlation coefficient

	H_1_				H_2_				H_1_ vs H_2_	
	95 % CI		p	r	95 % CI		p	r	p	
	ECG	PPG			ECG	PPG			ECG	PPG
mHR	83.09–85.59	83.19–85.64	*0.002*	1.00	86.29–93.13	86.64–93.32	*0.016*	0.99	*0.000*	*0.001*
sdHR	5.99–6.55	4.35–5.11	*0.000*	0.90	6.66–7.97	5.11–5.99	*0.000*	0.92	*0.000*	*0.000*
LTI	10.53–11.67	8.13–9.19	*0.000*	0.92	12.37–14.33	9.19–11.31	*0.000*	0.90	*0.000*	*0.000*
Delta	21.42–23.33	10.16–11.44	*0.000*	0.71	24.19–27.13	11.30–13.78	*0.000*	0.81	*0.000*	*0.000*
STV	3.53–3.89	0.94–1.03	*0.000*	0.60	3.85–4.41	1.05–1.18	*0.000*	0.71	*0.001*	*0.000*
II	0.56–0.63	0.19–0.21	*0.000*	0.74	0.55–0.60	0.18–0.21	*0.000*	0.72	0.177	0.573
TP	25.82–30.27	11.42–14.32	*0.000*	0.83	33.50–44.59	14.05–21.23	*0.000*	0.89	*0.000*	*0.000*
VLF	8.53–10.57	7.53–9.49	*0.000*	0.95	11.13–15.57	8.88–13.56	*0.000*	0.94	*0.000*	*0.001*
LF	6.67–8.12	3.20–4.01	*0.000*	0.88	8.16–10.87	4.14–5.47	*0.000*	0.90	*0.000*	*0.000*
^LF^norm	45.28–50.38	90.90–92.56	*0.000*	0.44	43.29–49.99	91.69–92.76	*0.000*	0.41	0.926	0.643
HF	4.70–5.61	0.16–0.20	*0.000*	0.71	5.90–7.69	0.21–0.27	*0.000*	0.73	*0.000*	*0.000*
^HF^norm	28.33–32.18	4.70–5.49	*0.000*	0.45	28.03–32.48	4.66–5.55	*0.000*	0.45	0.805	0.958
LF/HF	1.47–1.69	16.67–19.56	*0.000*	0.48	1.35–1.67	16.48–19.73	*0.000*	0.46	0.944	0.981
ApEn (2,0.1)	1.21–1.24	0.55–0.60	*0.000*	0.23	1.16–1.21	0.57–0.61	*0.000*	0.22	*0.010*	0.276
ApEn (2,0.15)	1.15–1.20	0.52–0.56	*0.000*	0.43	1.10–1.15	0.48–0.56	*0.000*	0.47	*0.023*	0.371
ApEn (2,0.2)	0.98–1.04	0.26–0.45	*0.000*	0.51	0.96–1.02	0.24–0.34	*0.000*	0.53	0.163	0.620
SampEn (2,0.1)	1.52–1.62	0.38–0.45	*0.000*	0.38	1.41–1.54	0.40–0.46	*0.000*	0.59	*0.039*	0.524
SampEn (2,0.15)	1.16–1.29	0.33–0.39	0.000	0.38	1.06–1.16	0.31–0.38	*0.000*	0.46	*0.001*	0.336
SampEn (2,0.2)	0.91–0.99	0.20–0.28	*0.000*	0.51	0.87–0.96	0.18–0.22	*0.000*	0.58	0.062	0.479
rLu	0.13–0.13	0.06–0.06	*0.000*	0.48	0.12–0.13	0.06–0.06	*0.000*	0.36	0.074	0.286
ApEn (2,rLu)	1.20–1.24	0.57–0.61	*0.000*	0.18	1.15–1.21	0.60–0.63	*0.000*	0.12	*0.007*	*0.003*
SampEn (2,r_Lu_)	1.33–1.39	0.40–0.46	*0.000*	0.08	1.22–1.32	0.43–0.49	*0.000*	0.07	*0.000*	0.052
SD1	1.57–1.74	0.37–0.39	*0.000*	0.47	1.68–2.17	0.40–0.46	*0.000*	0.44	*0.004*	*0.000*
SD2	8.21–9.06	6.13–7.22	*0.000*	0.91	9.17–10.94	7.21–8.46	*0.000*	0.93	*0.000*	*0.000*
SD1/SD2	0.19–0.21	0.06–0.06	*0.000*	0.48	0.17–0.19	0.05–0.06	*0.000*	0.36	0.078	0.308

The last two columns display the p-values of the comparison between H_1_ and H_2_

p-values <0.05 are in italics

Progression of labor (from H_1_ to H_2_) was associated with a significant increase in SD_1_, SD_2_ and most linear indices (excluding II, LF_norm_, HF_norm_ and LF/HF) and with a decrease in entropy indices (Table 2). This was generally observed with both acquisition modes, with the exception of most entropy-based indices in PPG signals.

**Fig. 3:**
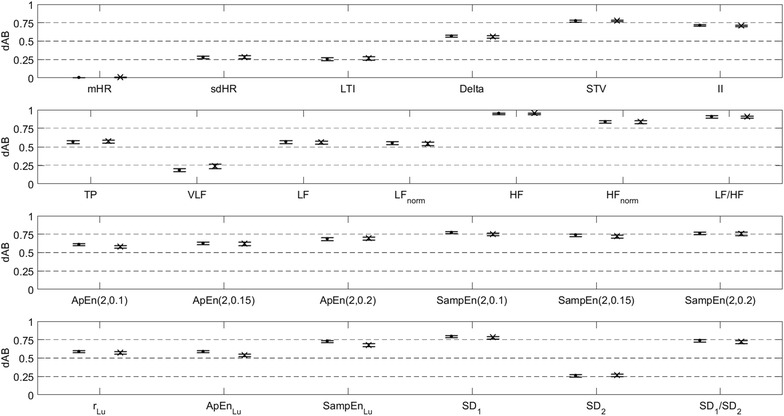
Disagreement (d_AB_) between the MHR variability indices obtained with ECG and PPG, for linear time-domain indices (*first row*), linear frequency-domain indices (*second row*), entropy indices (*third row*) and Poincaré plot indices (*fourth row*). Disagreement was assessed for the initial (H_1_, *dot*) and final (H_2_, *cross*) 10-min tracing segments (*error bars* represent the 95 % CI). Note that the zero value corresponds to the minimum disagreement (maximum agreement)

MHR indices related with short-term variability or faster oscillations had the highest disagreements between ECG and PPG recording modes, namely the linear time-domain indices Delta, STV and II, the linear frequency-domain indices, excluding VLF, and the non-linear indices, excluding SD_2_, for which d_AB_ was in the range 0.57–0.96 (Fig. 3).

**Fig. 4:**
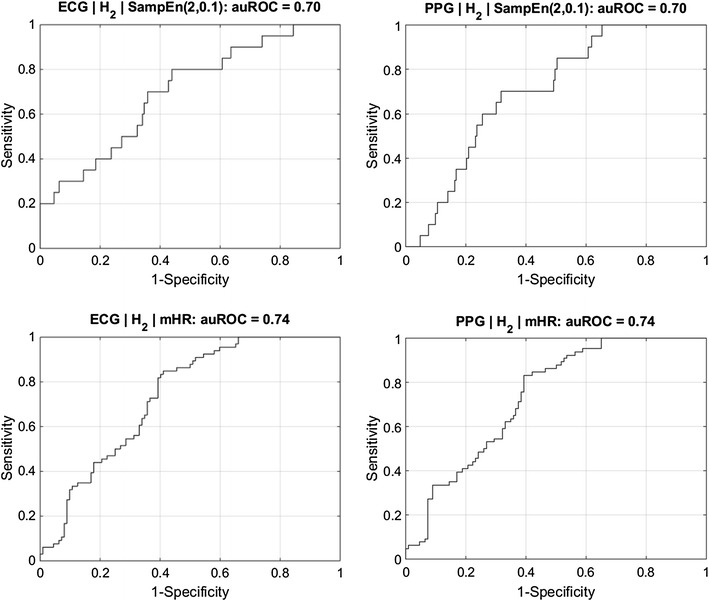
Receiver operating characteristic curves for the detection of fetal acidemia in H_2_ (*top plots*) using SampEn(2,0.1), with ECG (*left plot*) and PPG (*right plot*), both associated with an auROC of 0.70. A similar analysis for the detection of operative delivery is given in the lower plots, for which an auROC of 0.74 was achieved using mHR in H_2_

Considering the analysis of normal versus acidemic newborns, MHR variability indices obtained with ECG or PPG were not significantly different between both groups in H_1_. However, there were significant differences in H_2_, both with ECG and with PPG. The highest auROC values were obtained with SampEn(2,0.1) in H_2_, with similar auROC values of 0.70 for ECG and PPG (Fig. 4).

There were also significant differences between normal and vaginal operative deliveries in several MHR indices in H_1_ and H_2_, both with ECG and PPG signals. The highest auROC value was 0.74, obtained with mHR in H_2_ for ECG and PPG (Fig. 4).

## Discussion

To our knowledge, this is the first study to compare linear and non-linear MHR variability indices obtained with ECG and PPG during labor. The main objective was to determine whether PPG can be used as an alternative to ECG, as the latter may not always be available.

The clinical scenarios chosen to perform our comparison between MHR variability indices derived from signals obtained with ECG or PPG were progression of labor, fetal acidemia and operative vaginal delivery, as these are conditions related with different maternal balances between cortical and autonomic nervous system activities (Tejera et al. 2011; Task force of the European Society of Cardiology, the North American Society of Pacing and Electrophysiology 1996).

The percentage of signal loss with the PPG signal was similar to that of the ECG during the final segments of labour, and was only significantly higher in the last 10-min segment (S_6_) of H_2_. This may have happened because of maternal movements and inadvertent temporary removal of the pulse oximeter in this more stressful period. In addition, the percentage of signal loss of the PPG signal was significantly lower than the ECG in the first four segments of H_1_. Therefore, the use of PPG is not compromised because of signal loss in the last 2 h before delivery, with the exception of the final 10-min segment.

The similarity of MHR variability indices calculated with ECG and PPG signals was not the same for all indices. Those related with faster oscillations and entropy were poorly correlated and exhibited high disagreement. Linear indices related with baseline and long-term variability (e.g. mHR, VLF and SD_2_) were highly correlated and had low disagreement. These findings corroborate the different visible characteristics of the ECG and PPG signals (Fig. 1).

Visual analysis of the PPG signal suggests an absence of the high frequency component, supported by considerably lower absolute values of HF than the ECG signal. Although to a lesser extent, TP, VLF and LF indices derived from the PPG signal were also lower than their ECG counterparts. LF_norm_ was approximately twice, HF_norm_ was smaller to a larger extent and LF/HF was much higher in PPG signals. In spite of this, a consistent trend from H_1_ to H_2_ was observed with both ECG and PPG signals (Table 2). We can therefore conclude that, although spectral indices derived from the two signals are significantly different, if appropriate reference ranges are used, PPG signals may be used as an alternative to the ECG for traditional HRV spectral analysis.

A possible explanation for the difference between ECG and PPG signals is related with the HR extraction technique. A beat detection approach is usually employed in the ECG signal, whereas spectral analysis is commonly used with PPG signals. The narrower shape of the peak in the ECG signal may lead to a higher resolution. Unfortunately, access to the raw PPG signal data was not possible in this study, nor could detailed information on this extraction technique be obtained from the manufacturers. It should be noted that the differences between ECG and PPG signals may also be related to the inherently different nature of these signals: the PPG signal depends on the detection of blood ejected from the heart measuring changes in light absorption, whereas ECG signals evaluate electrical activity of the heart. These two signals should naturally have the same periodicity for healthy subjects, but they can differ in subjects with cardiac dysrhythmias. Patients with atrio-ventricular block or atrial fibrillation can exhibit an electro-mechanical cardiac dysynchrony. While these situations are unlikely to occur in a population of healthy women with uneventful pregnancies, such as the one in the present study, they may appear in higher-risk pregnancies. Bizarre and noisy MHR signals obtained by the ECG are typically associated with the most significant and persistent cardiac disrythmias (Cabaniss 1993).

Progress of labor was associated with an increase of most MHR linear indices and a decrease of entropy indices, with both acquisition methods. This is consistent with a previously described increase in autonomic nervous system activity throughout labor, both from the maternal and fetal sides (Pinto et al. 2014; Gonçalves et al. 2006b). PPG signals seem to be as good as ECG ones for establishing the evolution of slow oscillation-based MHR indices throughout labour.

Similar auROC values were obtained in the discrimination between acidemic and normal fetuses, and between normal and vaginal operative deliveries, when using ECG and PPG signals. The highest observed value of 0.70 using SampEn(2,0.1) in H_2_ regarding fetal acidemia, and of 0.74 using mHR in H_2_ in the detection of operative vaginal delivery, opens the possibility of single or combined use of MHR and FHR indices, in the identification of these situations. The discriminatory capacity of these indices may be higher for detection of fetal acidemia when considering a multivariate approach (Gonçalves et al. 2016). However, the objective of maximizing discriminatory performance was not under the scope of the present study.

Further refinement of pre-processing and processing algorithms may optimize the results reported in this study, regarding clinical applications. Conventional fetal monitors supply MHR at 4 Hz intervals, whereas the frequency bands of interest in human adults are in the range 0–0.4 Hz. Accordingly, 80 % of the spectrum corresponding to the interval between 0.4 Hz and the Nyquist frequency (2 Hz) is of little interest. Therefore, signal acquisition or resampling at 2 Hz rather than 4 Hz may be considered, as in the present study, and this may allow a reduction in computation time without compromising results. However, particular care must be taken when analyzing MHR signals at other untested sampling frequencies (smaller than 2 Hz or beat-to-beat), as this has been shown to influence the results of variability indices (Gonçalves et al. 2013). Additionally, the sampling frequency, presence of noise and the filtering procedure of the original signal, and different equipment from the one considered in this study, must be carefully evaluated.

## Conclusion

In conclusion, although PPG capture faster oscillations of the MHR signal less well than ECG and is prone to have higher signal loss in the last 10-min preceding delivery, it can be considered an alternative for MHR monitoring during labour, when appropriate MHR variability indices are used with a proper adaption of cut-off intervals. Further studies are warranted to confirm whether access to the PPG raw signal data or more detailed information on the extraction method will improve performance, and whether linear and entropy analysis of MHR, alone or in combination with FHR, may be clinically useful.
